# Sonication of Vascular Grafts and Endografts to Diagnose Vascular Graft Infection: a Head-To-Head Comparison with Conventional Culture and Its Clinical Impact

**DOI:** 10.1128/spectrum.03722-22

**Published:** 2023-02-27

**Authors:** Lisanne Braams, Gro Vlaspolder, Kathleen Boiten, Elisa Salomon, Rik Winter, Ben Saleem, Marjan Wouthuyzen-Bakker, Marleen van Oosten

**Affiliations:** a Department of Medical Microbiology and Infection Prevention, University of Groningen, University Medical Center Groningen, Groningen, the Netherlands; b Division of Vascular Surgery, Department of Surgery, University of Groningen, University Medical Center Groningen, Groningen, the Netherlands; University of Texas Southwestern Medical Center

**Keywords:** culture methods, diagnostics, sonication, vascular graft infection

## Abstract

Vascular graft and endograft infection (VGEI) is a severe complication associated with high mortality and is often challenging to diagnose. For the definitive microbiological diagnosis, sonication of vascular grafts may increase the microbiological yield of these biofilm-associated infections. The objective of this study was to determine whether sonication of explanted vascular grafts and endografts results in a higher diagnostic accuracy than conventional culture methods and aids in clinical decision-making. A prospective diagnostic study was performed comparing conventional culture with sonication culture of explanted vascular grafts in patients treated for VGEI. Explanted (endo)grafts were cut in halves and were either subjected to sonication or conventional culture. Criteria based on the Management of Aortic Graft Infection Collaboration (MAGIC) case definition of VGEI were used for definitive diagnosis. The relevance of sonication cultures was assessed by expert opinion to determine the clinical impact on decision-making. Fifty-seven vascular (endo)graft samples from 36 patients (four reoperations; 40 episodes) treated for VGEI were included; 32 episodes were diagnosed with VGEI. Both methods showed a positive culture in 81% of the cases. However, sonication culture detected clinically relevant microorganisms that went unnoticed by conventional culturing in 9 out of 57 samples (16%, 8 episodes) and provided additional relevant information regarding growth densities in another 11 samples (19%, 10 episodes). Sonication of explanted vascular grafts and endografts improves the microbiological yield and aids in the clinical decision-making for patients with a suspected VGEI compared to conventional culture alone.

**IMPORTANCE** Sonication culture of explanted vascular grafts was shown to be a noninferior method compared to conventional culturing in diagnosing vascular graft and endograft infection (VGEI). Moreover, sonication culture has probable additional value in microbiological characterization of VGEI by giving more detailed information on growth densities, especially when the conventional culture shows intermediate growth. In this prospective design, for the first time, a direct comparison is made between sonication culturing and conventional culturing in VGEI, while taking clinical interpretation into account. Therefore, this study is another step toward a more accurate microbiological diagnosis of VGEI, influencing clinical decision-making.

## INTRODUCTION

Vascular graft and endograft infections (VGEIs) are a severe complication in vascular surgery, occurring in 1 to 6% of cases (1). Even though its occurrence is rare, VGEI is associated with high morbidity and has a mortality of up to 88% (2). Diagnosing VGEI is challenging, as blood cultures are often negative (3–7), and a “gold standard” diagnostic test is lacking (7, 8). The treatment of VGEI comprises surgical graft replacement followed by an extensive antibiotic treatment of several weeks to months.

Criteria for suspecting VGEI, as proposed by Fitzgerald et al. (7), are based on microbiological culturing, radiological characteristics, increased inflammatory markers, and clinical symptoms. The Management of Aortic Graft Infection Collaboration (MAGIC) produced a definition in a process of expert review and consensus for aortic graft infection (8). However, these criteria still need to be translated to peripheral prosthetic graft infections and dialysis arterio-venous shunts. Usually, the final confirmation of VGEI diagnosis follows from (partly) explanting and subsequently culturing the vascular graft together with culturing of periprosthetic tissue. An accurate diagnosis of VGEI and determination of causative microorganisms, including antibiotic susceptibility testing, are essential for the antimicrobial treatment options and overall success rate of treatment. The procedure of graft replacement is not only mandatory for diagnosis in many cases but also indispensable for treatment, as an infected vascular graft cannot be cured with antimicrobial treatment alone (2). In the unfortunate case that curative treatment is failing, a conclusive microbiological diagnosis is also of major importance to tailor suppressive therapy options.

As is known from the field of orthopedic infections and other implant-associated infections, microbiological culturing of implants is hampered by biofilm formation of the causative pathogens, which leads to a lower culture sensitivity and may result in false-negative culture results (9–11). It has been shown that by sonicating medical implants before culturing, ultrasonic waves can disrupt the biofilm and increase the microbiological yield and sensitivity of culturing (9, 10). Of note, two studies recently have investigated the impact of sonication on vascular graft culture (5, 12).

Therefore, the objective of this study was to determine whether sonication of explanted vascular prosthetic grafts results in a higher diagnostic yield than conventional culture of vascular grafts and aids in establishing the microbiological diagnosis and clinical decision-making in VGEI.

## RESULTS

### Study population.

Thirty-six patients were included, including four patients with reoperation, adding up to 40 suspected VGEI episodes (Table 1). Thirty-two episodes were diagnosed as VGEI; in 8 episodes, VGEI diagnosis was rejected. A total of 57 samples were retrieved, as grafts were occasionally delivered in multiple parts, where each part counted as an individual sample.

**TABLE 1:** Descriptive statistics

Descriptive statistics	Numbers
Patients	
No. of patients	36
Reoperation samples	4
Infection episodes (including reoperation)	40
Male	29
Female	7
Age (median; range)	67; 41 to 84
Culture results^*a*^	
No growth	13
Monomicrobial infection	11
Polymicrobial infection (>1 m.o. in conventional culture)	16
Antibiotic use 48 h before surgery^*a*^	25/40 (63%)
Grafts^*a*^	
Samples	57
No growth	22/57 (40.4%)
Monomicrobial	16/57 (28.1%)
Polymicrobial (>1 m.o. in conventional culture)	19/57 (31.6%)
Anatomical site^*a*^	
Aortabifurcation/-bifemoral	23
Iliofemoral	3
Crossover	5
Fem-pop	4
Dialysis shunts	3
Patch	2
No. of m.o. conventional culture (median; interquartile range)	2; 1 to 3
No. of m.o. sonication culture (median; interquartile range)	2; 1 to 3
Preoperative/intraoperative cultures	
Patients with blood cultures	28/40 (70%)
Sets per patient (avg)	3
Patients with positive blood cultures	10/28 (36%)
Match m.o. graft with total blood culture m.o.	12/15 (80%)
Patients with intraoperative cultures	17/40 (43%)
Match m.o. graft with total intraoperative culture m.o.	68/98 (69%)
Extra relevant m.o. found in intraoperative cultures	30/98 (31%)

a Four patients were operated on twice, and some patients had multiple grafts. Therefore, totals add up to more than 36 patients depending on the analysis. Contamination was excluded. No., number; m.o., microorganisms; fem-pop, femoral-popliteal; avg, average.

Most patients had abdominal grafts (23/40 episodes), followed by peripheral vascular grafts (12/40 episodes). Of the positive samples (35/57, 28 episodes), 54% showed polymicrobial growth (19/35 samples, 16 episodes). One patient had a Coxiella burnettii infection, resulting in secondary infection by gastrointestinal flora through an aorto-duodenal fistula.

In most cases, blood cultures (28/40 episodes, 70%) were drawn preoperatively, of which 36% (10/28 episodes) were positive. In the positive blood cultures, a total of 15 microorganisms were identified, matching the cultured graft microorganisms in 80% (12/15). In 43% (17/40 episodes), intraoperative deep tissue cultures were collected, matching the cultured graft microorganisms in 69% (68/98) (Table 1).

According to local guidelines, in 25 episodes (34/57 samples), antimicrobial treatment was given 48 h before surgery; 11 patients received the standard regimen of piperacillin-tazobactam, vancomycin, and caspofungin. Antimicrobial treatment seemed to not have a significant influence on culture (*P* = 0,31); 7/25 patients who received antibiotics had positive culture results (with microorganisms susceptible for the administered antibiotics) compared to 7/15 patients without antibiotics.

### Cultured microorganisms.

Monomicrobial samples (*n* = 11) (Fig. S2A and B in the supplemental material) showed mainly skin flora, such as Staphylococcus aureus and coagulase-negative staphylococci (CNS), while polymicrobial samples showed a myriad of gastrointestinal aerobic and anaerobic flora (Fig. S2C and D). Accordingly, polymicrobial samples (18/57 samples) were mostly retrieved from aortobifurcation/bifemoral grafts (67%, 12/18 samples); only one polymicrobial infection with skin flora was observed in a peripheral femoral-popliteal (fem-pop) graft (Table S1). Notably, yeasts were cultured in 10/57 samples (7/40 episodes) generally retrieved from abdominal grafts (9/10 samples).

### Sonication versus conventional culture.

Both methods were tested on diagnostic performance (Table 2). Both conventional and sonication culture diagnosed infection in 81% (26/32 episodes) of the episodes that were marked as VGEI by the multidisciplinary team using the MAGIC criteria as stated in the methods, section case definition of VGEI. In 6 episodes, both culture methods remained negative while VGEI was diagnosed; local clinical signs of infection in combination with a positive positron emission tomography (PET) scan was the most frequent reason to classify VGEI in these 6 episodes. For sonication culture results, a cutoff value of <5 colony forming units (CFU) was used to exclude contamination. This cutoff was proposed by Trampuz et al. in sonication of prosthetic joint infections and was enforced by Gomes et al. in infective endocarditis (9, 10).

**TABLE 2:** Diagnostic performance^*c*^

	Episodes (*N* = 40)		Grafts (*N* = 57)		Cultured causative microorganisms (*N* = 107) and contaminants (*N* = 26)	
Method	Positive results/VGEI^*a*^	Negative results/non-VGEI^*a*^	Positive results/VGEI^*a*^	Negative results/non-VGEI^*a*^	Causative m.o./total causative m.o.	Contamination rate (contaminants/total m.o. per method)
Conventional culture	26/32	5/8	34/44	8/13	90/107	15/105 (14%)
Sonication culture	26/32^*b*^	7/8^*b*^	33/44^*b*^	13/13^*b*^	71/107^*b*^	11/82 (13%)^*b*^

a Total of 40 (non)infection episodes of 36 patients, including 4 reoperations.

b Contaminants excluded; cutoff value of <5 CFU.

c N, number; VGEI, vascular graft and endograft infection; m.o., microorganisms.

On the sample level, each species was reviewed by a medical microbiologist (M.v.O.), from which contamination rate per method was calculated. Sonication culture had an identical contamination rate as conventional culture; 13% and 14%, respectively (*P* = 0,51) (Table 2). However, it was easier to discriminate contamination from likely causative pathogens in the sonication method, as contamination grew in 9/11 cases ≤5 CFU, while causative pathogens grew in 58/71 cases >5 CFU. An exception was *Candida* species that repeatedly (5/12 cases) grew in ≤5 CFU but was considered a causative pathogen. In conventional culture, identifying contamination based on growth density was not feasible, as causative pathogens regularly grew at the same growth densities.

**FIG 1:**
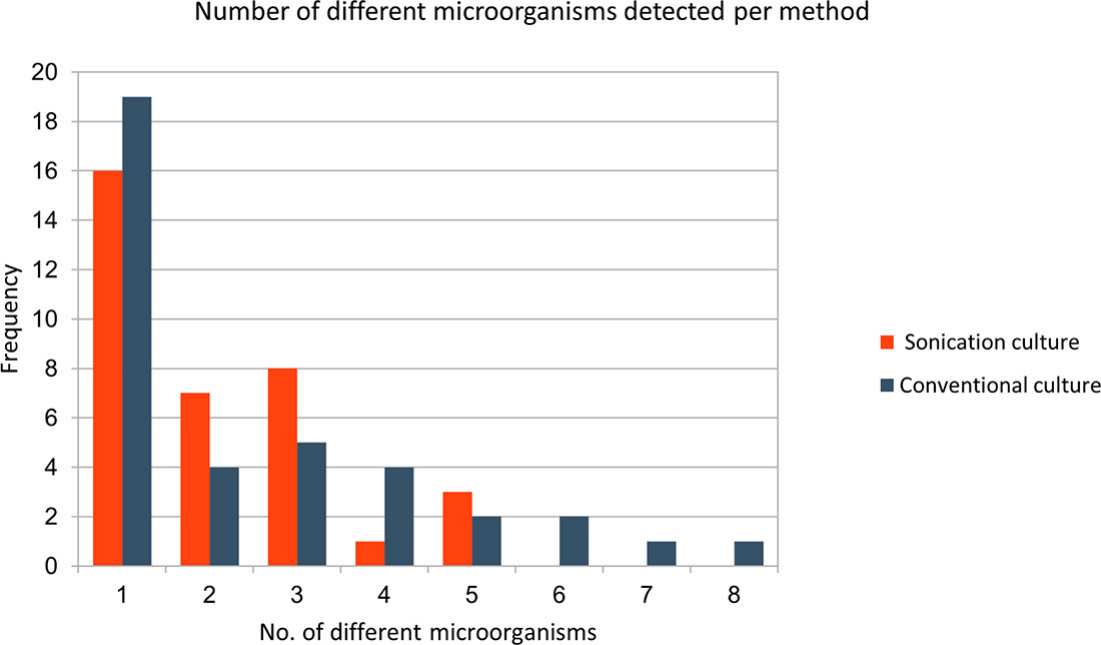
Number of different microorganisms detected per method. On the *y* axis, the frequency represents the number of cultured grafts, and on the *x* axis, the number of different microorganisms per sample is shown. Contamination was excluded. Of note, the conventional culture method was better able to detect a high species diversity per culture than the sonication method, in particular in anaerobic culturing. No., number.

We observed specifically in anaerobic cultures that solid agars tended to overgrow due to the plating technique, impeding discrimination and isolation of different anaerobic species. This is reflected in the higher number of microorganisms that was detected by conventional culturing (Fig. 1).

When directly comparing both methods in the same samples that showed clinically relevant growth, a complete match of the cultured species was found in 50% (54/107 of all cultured relevant species) (Table S2). In 32% of the mismatches on the species level (17/53 of all mismatched microorganisms), the conventional culture missed clinically relevant microorganisms. In the other 68% of mismatches (36/53 mismatches), the sonication culture missed clinically relevant microorganisms compared to conventional culture, of which 20 were anaerobic bacteria. Through sonication culture, S. aureus was found with high growth densities in cases 20 and 21 (two samples derived from the same infection episode) and was not identified by conventional culturing (case 20) or was solely identified in a low density in liquid medium (case 21). Similarly, Enterococcus faecalis (case 21), lactobacilli (case 6 and 34), and Eikenella corrodens (case 47) were detected by the sonication method and not by the conventional method.

### Growth density.

**FIG 2:**
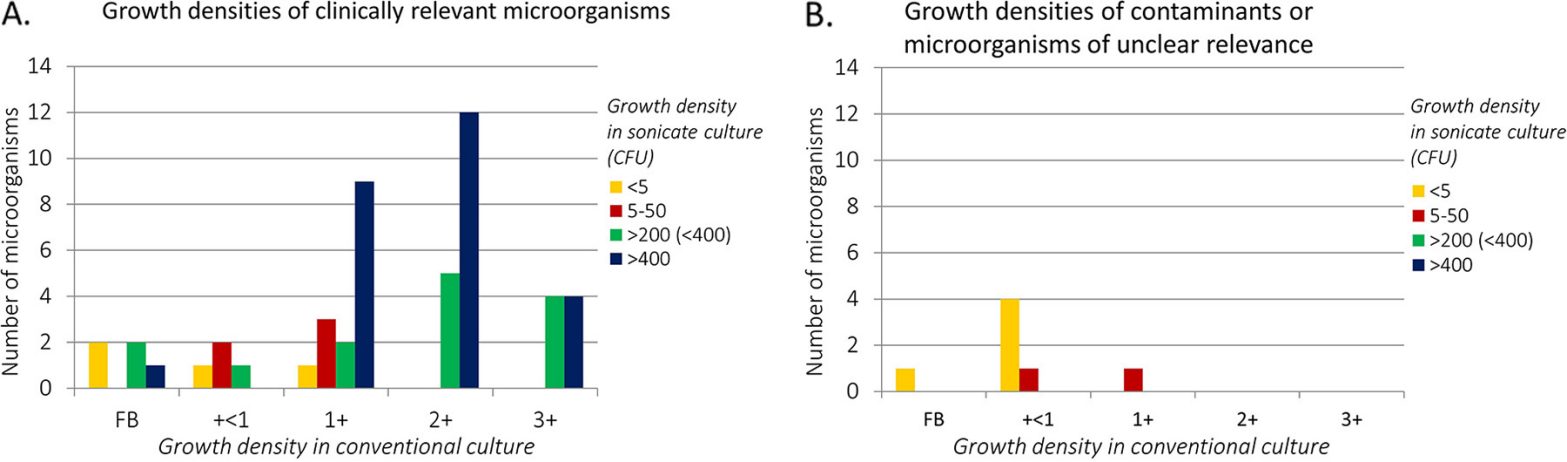
(A and B) Growth density comparison of microorganisms found in both the conventional culture and corresponding sonication culture for relevant microorganisms (A) and contaminants or microorganisms of unclear relevance (B). Samples with the same microorganism found in both conventional culture and sonicate culture were included in this analysis. On the *x* axis, the conventional culture growth density is displayed. FB represents no growth on solid agars but positive liquid culture, +<1 represents 1 to 10 CFU on solid agar, +1 is growth in the first segment of more than 10 CFU, +2 is growth in the second segment, and +3 is growth in the third segment. The sonication culture growth density is measured in CFU/100 μL, displayed in different colors; <5 CFU/100 μL (yellow); 5 to 50 CFU/100 μL (red); 200 to 400 CFU/100 μL (green); >400 CFU/100 μ L (blue). Of note, in grafts explanted in separate parts, the same microorganism found in different parts showed different growth densities, most likely due to an uneven distribution of the biofilm over the graft. (A) Microorganisms that were interpreted as relevant but had low growth densities in both methods (yellow) included two *Candida* species, one Klebsiella oxytoca, and one Cutibacterium acnes. *Candida* species were regularly found in low growth densities in both culture methods (in sonicate culture, ≤5 CFU) and were always considered relevant microorganisms. (B) Contaminants in conventional culture were included to determine whether sonication culture would also display a low growth density. The two contaminant microorganisms appointed to the category “growth density of 5 to 50 CFU in sonicate” (red) grew with a relatively low growth density of 5 and 15 CFU. CFU, colony forming units.

A head-to-head comparison was performed to assess whether sonication culture results in higher culture yield (Fig. 2). In most cases (40 species, 35 episodes), quantification in both methods correlated well; that is, fastidious broth (FB) and +<1 segmental growth corresponded with low CFU counts, and +2 and +3 segmental growth corresponded with high CFU counts. Microorganisms that grew at +1 segmental growth density (*n* = 16) showed a variety of CFU counts, where the CFU count of the sonication method seemed more reliable.

### Expert opinion on clinical decision-making.

Impact on clinical decision-making of the sonication results was scored (Table 3). Sonication detected clinically relevant microorganisms that went unnoticed by conventional culturing in 9 samples (16%, 8 episodes) (Table S2). However, the additional value of sonication was also observed in 11 out of 57 samples (19%, 10 episodes), where the results of sonication reenforced those of the conventional culture, the latter being doubtful when seen on their own.

**TABLE 3:** Expert opinion on clinical relevance

Category^*a*^	Frequency
1. Yes, clinically relevant microorganism is found in the sonicate culture, not in the conventional culture	9
2. Yes, the sonicate culture results enforces the conventional culture results	11
3. No, the sonicate culture and the conventional culture are consistently negative	19
4. No, the sonicate culture and the conventional culture are consistently positive	13
5. No, the microorganism found in the sonicate culture is not clinically relevant	5

a Two experts on VGEI (an infectious disease specialist and a medical microbiologist) independently scored the samples and answered the posed question, “Does sonication culturing have an additional clinical value to conventional culturing in this sample?”

## DISCUSSION

This study shows the noninferiority and probable additional value of sonication in microbiological characterization of VGEI compared to conventional culture, with positive impact on clinical decision-making.

Sonication is already an established method in the microbiological diagnostic workup of prosthetic joint infections (9, 13–18). Trampuz et al. (9) showed that culture of microorganisms of the removed implant is more sensitive than tissue culture and that the microbiological yield and sensitivity of culturing are increased when the biofilm of the prosthesis is disrupted by sonication, in particular in patients receiving antibiotics before surgery. Moreover, sonication is expected to provide more homogeneous inoculation of the culture medium than the rolling technique on solid agars used by most laboratories for graft culture.

Relatively few studies investigated the use of sonication for microbiological diagnostic purposes in VGEI. Over 30 years ago, sonication culture showed increased microbiological yield in canine models of Staphylococcus epidermidis VGEI (19, 20), and recently, two studies investigated the effect of sonication on vascular graft cultures in humans (5, 12). Puges et al. reported a sensitivity and specificity of sonicate fluid culture of 89.7% and 100%, respectively (*n* = 39), and no statistical difference between performances of conventional culture with and without sonication and genus-specific PCR (12). Ulcar et al. (5) compared sonication culture and broad-range PCR of the vascular graft to intraoperative tissue sample and blood culture results. Sonication culture identified the causative microorganism in 79.2% of cases (19/24 cases) and broad-range PCR in 66.7% (16/24 cases), with concordant results between sonication culture and broad-range PCR in only 29.2% (7/24 cases). Nonetheless, it needs to be noted that both studies do not take contamination rates into account, nor did there seem to be a clinical interpretation of the culture results.

In this study, the proportion of polymicrobial infections was relatively high (37%), which has been reported before (5, 21, 22). Polymicrobial infections were mostly seen in abdominal grafts and usually showed gastrointestinal flora. Sonication appeared to detect less different anaerobic bacteria species per culture, which is likely due to overgrowth of fast-growing species on the agar plates. We suggest taking this aspect into account when sonicating vascular grafts, for instance by adding a three-segmented plating technique on solid agar in anaerobic culturing. We emphasize the importance of researching the most optimal solution to tackle this matter.

Even though 25 patients received antibiotics 48 h before surgery, we did not observe a significant impact on the culture results by any culturing method, as has been shown before by Legout et al. (3).

When looking at the clinical value of sonication as determined by expert opinion, the sonication method is mostly consistent and noninferior to conventional culturing but offers an additional value by giving more detailed information on growth densities, especially when the conventional culture shows intermediate growth. In selected cases, the effect of biofilm disruption through sonication was clearly observed (see Fig. 2A, green and blue). Inversely, in four patients, sonication showed no growth compared to low growth densities in conventional culture, confirming contamination in the latter. Overall, sonication culture allowed for a better discrimination between contaminants and true pathogens.

The strength of this study is the prospective design, and, for the first time, a direct comparison is made between sonication and conventional culturing in VGEI, while taking clinical interpretation into account.

Limitations of this study include a potential sample bias. The same microorganisms found in different parts of the same graft were documented with different growth densities, most likely due to uneven biofilm distribution over the vascular (endo)graft. However, efforts were taken to counter this bias prospectively by dividing the grafts over both methods. In our opinion, the approach in this study is the closest approximation to a direct clinical comparison as one can reach.

In this study, molecular diagnostics were not taken into account, although we do think that these can be an important addition to the microbiological workup in VGEI (23). In particular, molecular approaches that detect a broad range of bacteria (e.g., 16S targeted) and yeast (e.g., 18S targeted) with high sensitivity could be of additive value to perform on sonication fluid. Importantly, such an approach should not be limited to the detection of single causative species due to the polymicrobial nature of a vast account of VGEI (e.g., next-generation sequencing) (12). To the best of our knowledge, such an approach has not yet been published for VGEI diagnostics. Puges et al. use a targeted PCR method in which the variety of pathogens found are limited by the panel that is used (12). Ulcar et al. use broad-range 16S PCR and partly overcome the limitation of species-targeted PCR but do not elaborate on whether polymicrobial infections can be diagnosed (5). Moreover, by targeting 16S, yeast cannot be identified.

Finally, a low threshold for diagnostic efforts for rare and nonculturable pathogens can be recommended, also in already diagnosed polymicrobial VGEI, as demonstrated by the Q-fever case in this study.

In conclusion, in a head-to-head comparison with conventional culture alone, sonication of vascular (endo)grafts improves the microbiological culture yield and aids in the clinical decision-making for patients with a suspected VGEI.

## MATERIALS AND METHODS

### Case definition of VGEI.

In line with the MAGIC case definition (8), diagnosis of VGEI was based on microbiological, radiological, biochemical, and clinical criteria. Microbiological criteria included positive preoperative cultures, periprosthetic cultures, blood cultures, or intraoperative culture samples as well as a *Coxiella burnettii* anti-phase I IgG antibody titer of ≥1:800. The diagnosis VGEI was suspected if at least two of the MAGIC criteria were present, which could not be explained in any other way. The definitive diagnosis was mostly based on culture of the explanted prosthesis. All cases of suspected VGEI were discussed in a multidisciplinary VGEI team, consisting of a vascular surgeon, an infectious disease specialist, and a medical microbiologist.

### Patient population.

All explanted vascular grafts suspected of VGEI were prospectively included at the University Medical Center Groningen (UMCG), a tertiary medical center in the Netherlands. The samples were collected between July 2018 and December 2019, with a total of 57 samples from 36 patients. Reoperations were performed in 4 patients and were included as separate infection episodes. All types of grafts were included, biological (bovine pericardium), endograft, and synthetic. For each patient, demographic data, type of graft, and antibiotic therapy were analyzed.

### Specimen collection.

Due to surgical and technical reasons, a vascular graft of one patient was often explanted in multiple sections, which were treated as individual samples. Samples were transported in a sterile container and processed at the diagnostic laboratory of the Medical Microbiology Department. Each sample was cut in half, lengthwise or in ~1-cm rings alternately separated to create halves (Fig. S1 in the supplemental material). Both halves were subjected to either the conventional microbiological workup or the sonication protocol. In addition, all preoperative blood cultures (Bactec, BD diagnostics, Sparks, MD) and intraoperative cultures (i.e., thrombus, tissue, fluid, or pus in proximity of the graft) were processed according to laboratory standard operating procedures.

### Conventional (microbiological) workup of vascular grafts and endografts.

Conventional cultures were processed as routine diagnostic workups. One-half of the graft was rolled across different agar plates, and the inoculum was spread into three segments. Solid agars included blood agar (BA; containing 5% sheep blood), chocolate agar (CHOC), colistin-oxolinic acid blood agar (COB), blood agar + 5% sheep blood/aztreonam agar (BAZ), MacConkey agar number 3 with crystal violet (MC3), Sabouraud dextrose agar (SAB) for yeast culture, Brucella blood agar (BBA; containing 5% sheep blood), Brucella blood agar with kanamycin/vancomycin (BBKV), phenylethyl alcohol agar (PEA), and *Bacteroides* bile esculin agar (BBE; all obtained from Mediaproducts BV, Groningen, the Netherlands) inoculated in this order. After inoculation on solid medium, the vascular graft was transferred to liquid medium (fastidious broth [FB]). All solid media were incubated up to 9 days. BA, CHOC, BAZ, and COB agar plates were incubated at 35°C under aerobic conditions with 5% CO_2_. MC3 and SAB agar plates were incubated at 35°C under aerobic conditions with O_2_. BBA, BBKV, PEA, and BBE agar plates were anaerobically incubated at 35°C. FB was incubated at 35°C under aerobic conditions with O_2_ and inoculated on solid agar after either clouding occurred or blindly after 7 days. All cultured microorganisms were identified by matrix-assisted laser desorption ionization–time of flight mass spectrometry (MALDI-TOF MS) analysis (Microflex LT mass spectrometer or MALDI Biotyper SMART, Bruker Daltonik GmbH, Bremen, Germany). No technical discrepancies between inoculated media within one culture were reported.

### Sonication of vascular grafts and endografts.

The other half of the graft was placed in a sterile container (Gamma on 8 k-gray; incense article number 550676, Beldico, Duiven, the Netherlands) for sonication. The sonication protocol used has been previously described for sonication of heart valves in case of infective endocarditis (10). Briefly, before use, the outside of the sterile container was decontaminated with 70% ethanol. Sterile Ringer’s solution was added (at least 140 mL) until the sample was more than 90% submerged. The container with the sample was vortexed for 30 s (IKA Vortex Genius 3), decontaminated again with 70% ethanol, and placed in the sonication bath (BactoSonic ultrasonic bath BS14.2, Bandelin Electronic GmbH & Co. KG) for 1 min at 40 kHz and a power density of 100%. After sonication, the container was decontaminated and vortexed for another 30 s. Fifty milliliters of sonicate fluid was centrifuged at 2,500 × *g* (3,500 rpm) for 15 min (Hettich Rotina 46 R centrifuge, Gemini BV), and the supernatant was discarded, maintaining 2 mL of sediment. Aliquots of 100 μL from the sonication sediment were inoculated onto solid agar plates using sterile drigalski spatulas. In addition, two drops of sonication sediment were resuspended in FB. Types of media and culturing conditions of the solid and liquid media were identical as in the standard microbiological workup.

### Analysis by expert opinion.

The clinical impact on decision-making of the sonication results was assessed by expert opinion. Investigating authors (L.B. and G.V.), one clinical microbiologist (M.v.O.), and one infectious disease specialist (M.W.B.) specialized in VGEI consecutively and independently scored the sonication results on clinical relevance and additional value in one of five categories (Table 3). The discordant ratings were discussed between the experts, and a final consensus rating was assigned.

### Statistical analysis.

Descriptive statistics were performed with interquartile ranges using a Mann-Whitney *U* test. Contamination rate and antibiotic use were assessed using the Fischer’s exact test (two-tailed). *P* values of <0.05 were considered statistically significant.

### Ethics.

Permission for this study was obtained from the Medical Ethical Review Board Committee (METc 2021/048) of the UMCG. The study was performed with adherence to the relevant guidelines of this review board. The participants were checked by the opt-out patient consent protocol, and the data were treated pseudoanonymously.
