# Supplementary material for: Sonication of Vascular Grafts and Endografts to Diagnose Vascular Graft Infection: a Head-To-Head Comparison with Conventional Culture and Its Clinical Impact
Source: Microbiol Spectr. 2023 Feb 27;11(2):e03722-22. doi: 10.1128/spectrum.03722-22 (PMC10100911; doi:10.1128/spectrum.03722-22)
Supplement: Supplemental file 1 — Supplemental material. Download spectrum.03722-22-s0001.pdf, PDF file, 0.7 MB [file spectrum.03722-22-s0001.pdf]

SUPPLEMENTALS

Figure S1: Two methods of dividing vascular graft samples in representative halves; Vascular grafts were transported in a sterile container and were either cut in ~ 1cm rings (left), alternately separated, or cut in half over the length of the graft (right) as to create two equal halves. These methods of dividing were selected to minimize the potential bias of an uneven biofilm distribution over the graft.
The grafts were cut with sterile scissors under sterile circumstances. Both (alternately separated) halves were subjected to either the standard microbiological workup or the sonication protocol.

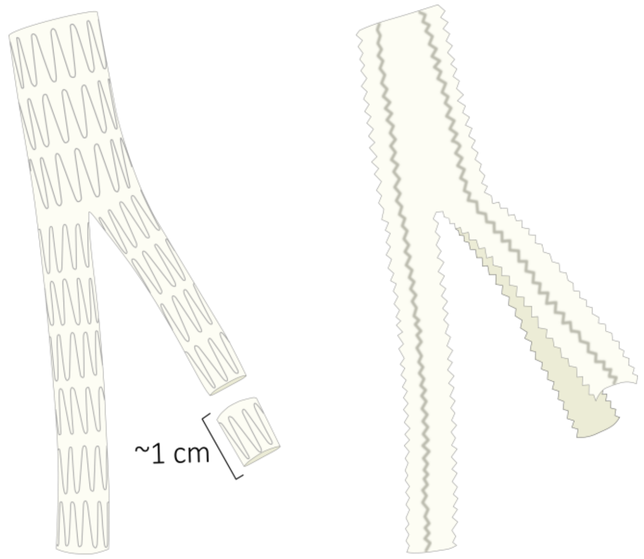

Figure S2 Proportion of detected microorganisms per method on sample level; conventional culturing (A,C) and sonication (B,D) further analyzed by monomicrobial (A,B) and polymicrobial (C,D) VGI. Polymicrobial was defined as >1 microorganism. Only positive samples were analyzed (35/57 samples) and contaminants were excluded.

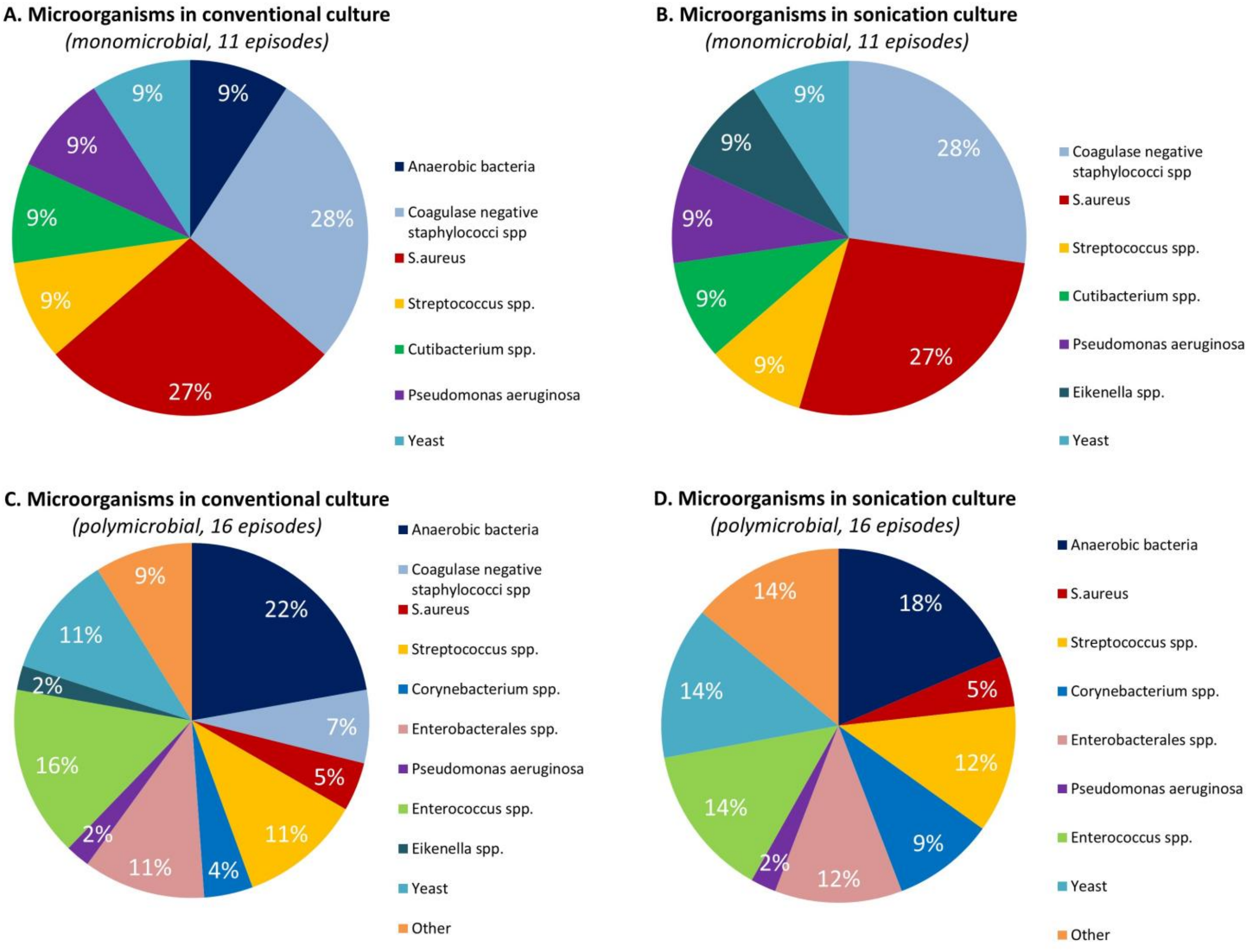

Table S1: Polymicrobial infections by type of graft and microbial flora

Type of graft	Number of polymicrobial samples (n=18/57)	GI flora*	Skin flora‡
**Aortabifurcation/-bifemoral**	12	12	0
**Crossover**	2	2	0
**Illiofemoral**	2	2	0
**Fem-pop**	1	0	1
**Patch**	1	1	0

*Gastrointestinal flora is defined as Gram-negative rods, anaerobic bacteria, streptococci, enterococci and/or yeast

‡Skin flora is defined as staphylococci, cutibacteriae and corynebacteriae

Table supplementary S2 Clinically relevant concordance of positive results; Comparison between conventional and sonication culture results of positive grafts (i.e. bacterial growth in one or both culture methods, excluding contaminants). Growth density of conventional culture was determined as following; FB represents no growth on solid agars, but positive liquid culture, +<1 represents 1-10 colony forming units (CFU) on solid agar, +1 is growth in the first segment of more than 10 CFU, +2 is growth in de second segment and +3 is growth in the third segment. The sonication growth density was measured in colony forming units (CFU) on solid agar. Below, concordance of both culture results is highlighted in green on species level, and discordance is presented in the number of (mis)matches on species level per graft. A summary of discordance across all grafts on species level is displayed at the end of the table. Contaminants, printed in grey, were excluded.

	Conventional culture			Sonication culture							
**Graft no.**	**Microorganism**	**Growth density (segments)**	**Clinical relevance**	**Microorganism**	**Growth density (CFU)**	**Clinical relevance**	**Total m.o. (excl. conta-minants)**	**Matches (excl. conta-minants)**	**Mismatches (excl. conta-minants)**	**Missed by Conven-tional culture**	**Missed by Sonication culture**
1	*Corynebacterium acnes*	FB	Relevant	*C. acnes*	1	Relevant	1	1	0	0	0
				Corynebacterium afermentans	4	Contamination					
				Corynebacterium tuberculostearicum	28	Contamination					
5	*Enterococcus faecium*	2+	Relevant	*E. faecium*	224	Relevant	6	3	3	2	1
	Klebsiella pneumoniae	<1+	Relevant	*K. pneumoniae*	1	Relevant					
	C. acnes	<1+	Contamination								
	Candida glabrata	1+	Relevant								
	C. albicans	FB	Relevant	*C. albicans*	1	Relevant					
				P. micra	2	Relevant					
				S. exigua	20	Relevant					
6	*Streptococcus anginosus*	3+	Relevant	*S. anginosus*	>400	Relevant	5	3	2	2	0
	Candida kefir	1+	Relevant	*C. kefir*	1	Relevant					
	Prevotella denticola	3+	Relevant	*P. denticola*	>400	Relevant					
				S. capitis	3	Contamination					
				Lactobacillus gasseri	>400	Relevant					
				Fusobacterium nucleatum	25	Relevant					
8	*Corynebacterium coyleae*	<1+	Unclear	*C. amycolatum*	1	Unclear	3	0	3	1	2
	Staphylococcus capitis	<1+	Unclear								
10	*E. faecium*	1+	Relevant	*E. faecium*	>200	Relevant	3	3	0	0	0
	Serratia marcescens	<1+	Relevant	*S. marcescens*	16	Relevant					
	C. albicans	2+	Relevant	*C. albicans*	>200	Relevant					

12	*Staphylococcus lugdunensis*	FB	Contamination				0	0	0	0	0
13	*S. lugdunensis*	FB	Contamination				0	0	0	0	0
14				*Staphylococcus hominis*	1	Contamination	0	0	0	0	0
15	*Pseudomonas aeruginosa*	1+	Relevant	*P. aeruginosa*	6	Relevant	3	1	2	1	1
	Propioni-bacterium avidum	<1+	Contamination	*P. avidum*	3	Contamination					
	Corynebacterium amycolatum	<1+	Contamination								
	Staphylococcus epidermidis	<1+	Contamination								
	Finegoldia magna	1+	Unclear								
				S. capitis	2	Contamination					
				P. hareii	1	Unclear					
19	*P. aeruginosa*	2+	Relevant	*P. aeruginosa*	>400	Relevant	1	1	0	0	0
	S. epidermidis	FB	Contamination	*S. epidermidis*	1	Contamination					
	Bacteroides fragilis	<1+	Contamination								
	Anaerococcus spp.	<1+	Contamination								
16	*S. epidermidis*	<1+	Relevant	*S. epidermidis*	10	Relevant	1	1	0	0	0
17	*E. faecium*	3+	Relevant	*E. faecium*	>200	Relevant	3	3	0	0	0
	C. albicans	2+	Relevant	*C. albicans*	>200	Relevant					
	C. glabrata	2+	Relevant	*C. glabrata*	>200	Relevant					
18	*Candida parapsilosus*	FB	Relevant				1	0	1	0	1
20	*S. epidermidis*	FB	Contamination				3	0	3	2	1
	Parvimonas micra	<1+	Unclear								
				Bacillus spp.	>200	Relevant					
				Staphylococcus aureus	>200	Relevant					
				Micrococcus luteus	18	Contamination					
21	*S. lugdunensis*	<1+	Unclear				7	1	6	1	5
	S. aureus	FB	Relevant	*S. aureus*	>200	Relevant					
	Peptoniphilus lacrimalis	<1+	Unclear								
	Peptococcus niger	<1+	Unclear								
	Fenollaria massiliensis	<1+	Unclear								

	Peptostreptococcus anaerobicus	FB	Unclear								
				E. faecalis	>200	Relevant					
22	*S. aureus*	<1+	Relevant	*S. aureus*	>200	Relevant	5	3	2	0	2
	Enterococcus faecalis	FB	Relevant	*E. faecalis*	>200	Relevant					
	C. amycolatum	<1+	Unclear	*C. amycolatum*	5	Unclear					
	S. lugdunensis	<1+	Unclear								
	P. micra	1+	Relevant								
30	*S. aureus*	2+	Relevant	*S. aureus*	>400	Relevant	5	1	4	2	2
	Anaerococcus spp.	1+	Relevant								
	F. magna	<1+	Relevant								
				Corynebacterium striatum	>26	Relevant					
				Dermabacter hominis	Not regis-tered	Relevant					
31	*Escherichia coli*	2+	Relevant	*E. coli*	>400	Relevant	4	1	3	1	2
	Lactobacillus spp	<1+	Relevant								
	Slackia exigua	1+	Relevant								
				S. anginosus	40	Relevant					
32	*Peptoniphilus hareii*	2+	Relevant	*P. hareii*	>400	Relevant	2	2	0	0	0
	Anaerococcus lactolyticus	1+	Relevant	*A. lactolyticus*	>400	Relevant					
33	*P. hareii*	2+	Relevant	*P. hareii*	>400	Relevant	2	2	0	0	0
	A. lactolyticus	2+	Relevant	*A. lactolyticus*	>400	Relevant					
34	*Streptococcus milleri group*	3+	Relevant	*Streptococcus milleri group*	>200	Relevant	10	3	7	2	5
	E. coli	3+	Relevant	*E. coli*	>200	Relevant					
	F. magna	3+	Relevant	*F. magna*	>200	Relevant					
	S. lugdunensis	1+	Relevant								
	E. faecalis	1+	Relevant								
	Lactobacillus crispatus	3+	Relevant								
	P. denticola	3+	Relevant								
	S. exigua	3+	Relevant								
				Granulicatella adiascens	>200	Relevant					
				Lactobacillus rhamnosus	>200	Relevant					
35	*K. pneumoniae*	1	Relevant	*K. pneumoniae*	>200	Relevant	2	1	1	0	1
	Group G Streptcoccus	<1+	Relevant								

36	*E. faecium*	2+	Relevant	*Enterococcus spp.*	>400	Relevant	4	3	1	0	1
	C. albicans	1+	Relevant	*C. albicans*	>400	Relevant					
	C. glabrata	1+	Relevant	*C. glabrata*	>400	Relevant					
	Candida tropicans	1+	Relevant								
37	*E. faecium*	3+	Relevant	*E. faecium*	>400	Relevant	5	2	3	1	2
	C. glabrata	1+	Relevant	*C. glabrata*	>400	Relevant					
	C. albicans	<1+	Relevant								
	Actinomyces turicensis	3+	Relevant								
				C. tropicans	4	Relevant					
38	*S. aureus*	1+	Relevant	*S. aureus*	>400	Relevant	1	1	0	0	0
39	*Proteus mirabilis*	1+	Relevant	*P. mirabilis*	34	Relevant	6	4	2	0	2
	E. faecalis	1+	Relevant	*E. faecalis*	400	Relevant					
	S. anginosus	1+	Relevant	*S. anginosus*	400	Relevant					
	P. denticola	2+	Relevant	*P. denticola*	>400	Relevant					
	Campylobacter gracilis	2+	Unclear								
	Bifidobacterium dentium	2+	Unclear								
40	*S. epidermidis*	1+	Relevant	*S. epidermidis*	>400	Relevant	1	1	0	0	0
41	*S. epidermidis*	<1+	Unclear	*S. epidermidis*	1	Unclear	1	1	0	0	0
				Staphylococcus warneri	2	Contamination					
42	*Staphylococcus haemolyticus*	<1+	Contamination				0	0	0	0	0
45	*S. anginosus*	1+	Relevant	*S. anginosus*	>400	Relevant	7	3	4	0	4
	Bilophila wadsworthia	1+	Relevant	*B. wadsworthia*	>400	Relevant					
	Campylobacter curvus	FB	Relevant	*C. curvus*	>400	Relevant					
	Prevotella oris	1+	Relevant								
	Eikenella Corrodens	FB	Relevant								
	F. nucleatum	FB	Relevant								
	Fusobacterium naviforme	FB	Relevant								
46	*P. avidum*	<1+	Contamination				1	0	1	0	1
	C. tuberculostearicum	<1+	Contamination								
	Anaerobic Gram positive rods	<1+	Unclear								
				Kocuria rhizophila	1	Contamination					
47	*S. anginosus*	2+	Relevant	*S. anginosus*	300	Relevant	2	1	1	1	0
				E. corrodens	10	Relevant					

48	*S. epidermidis*	<1+	Unclear	*S. epidermidis*	1	Unclear	1	1	0	0	0
49	*S. aureus*	1+	Unclear	*S. aureus*	15	Unclear	1	1	0	0	0
	Corynebacterium accolens	<1+	Contamination	*C. amycolatum*	2	Contamination					
	C. amycolatum	FB	Contamination								
52	*C. striatum*	2+	Relevant	*C. striatum*	>400	Relevant	6	2	4	1	3
	B. fragilis	3+	Relevant	*B. fragilis*	>400	Relevant					
	Peptoniphilus spp.	2+	Relevant								
	F. magna	2+	Relevant								
	E. faecium	FB	Unclear								
				C. glabrata	1	Unclear					
53	*S. aureus*	2+	Relevant	*S. aureus*	>400	Relevant	1	1	0	0	0
54	*S. aureus*	2+	Relevant	*S. aureus*	>400	Relevant	1	1	0	0	0
56	*C. albicans*	2+	Relevant	*C. albicans*	>400	Relevant	1	1	0	0	0
57	*C. albicans*	1+	Relevant	*C. albicans*	17	Relevant	1	1	0	0	0

Summary

**Total (mis)matches**	**Number of m.o.**
Matches (excl. contaminants)	54/107 (50%)
Mismatches (excl. contaminants)	53/107 (50%)
Missed by Conventional culture	17/53 (32%)
Missed by Sonication culture	36/53 (68%)

Table S3 Supplementary: Repeated surgeries

Of the four patients that underwent reoperation, the conventional culture and sonication results are compared. Contamination is printed in bold. Growth density of conventional culture is determined as following; +<1 represents 1-10 colony forming units (CFU) on solid agar, +1 is growth in the first segment of more than 10 CFU, +2 is growth in de second segment and +3 is growth in the third segment. The sonication growth density is measured in colony forming units (CFU) on solid agar.

Case no 1st	Case no 2nd	Time between revision	Culture 1st surgery	Growth density (segmental)	Sonication 1st surgery	Growth density (CFU)	Culture 2nd surgery	Growth density (segmental)	Sonicaton 2nd surgery	Growth density (CFU)
8	56;57	14 months	*Corynebacterium coyleae;*<br>*Staphylococcus capitis*	<1+<br><1+	*Corynebacterium amycolatum*	1	*Candida albicans*	2+	*Candida albicans*	>400
7	32;33	5 months	No growth		No growth		*Peptoniphilus hareii,*<br>*Anaerococcus lactolyticus*	2+<br>2+	*Peptoniphilus hareii,*<br>*Anaerococcus lactolyticus*	>400<br>>400
15	19	2 months	*Pseudomonas aeruginosa;*<br>***Cutibacterium avidum***<br>***Corynebacterium amycolatum;***<br>***Staphylococcus epidermidis;***	1+<br>**<1+**<br>**<1+**<br>**<1+**	*Pseudomonas aeruginosa;*<br>***Cutibacterium avidum;***<br>***Staphylococcus capitis;***<br>*Peptoniphilus hareii*	6<br>**3**<br>**2**<br>**1**	*Pseudomonas aeruginosa;*<br>***Staphylococcus epidermidis;***<br>***Bacteroides fragilis;***<br>***Anaerococcus spp.***	2+<br>**FB**<br>**<1+**<br>**<1+**	*Pseudomonas aeruginosa;*<br>***Staphylococcus epidermidis***	>400<br>**1**
35	42	9 months	*Klebsiella pneumoniae;*<br>*Streptococcus* group G	+1<br>+<1	*Klebsiella pneumoniae*	>200	*Staphylococcus haemolyticus*	+<1	No growth	
